# Impact of the Angles of the Septal Deviation on the Degree of the Mastoid Pneumatization

**DOI:** 10.22038/ijorl.2019.35284.2162

**Published:** 2020-05

**Authors:** Mustafa Çelik, Yakup Yegin, Burak Olgun, Ahmet Altıntaş, Fatma-Tülin Kayhan

**Affiliations:** 1 *Department of Otorhinolaryngology-Head and Neck Surgery, Kafkas University, Faculty of Medicine, Kars, Türkiye. *; 2 *Sadi Konuk Training and Research Hospital, Department of Otorhinolaryngology - Head and Neck Surgery, Istanbul, Turkey.*; 3 *Department of Otorhinolaryngology - Head and Neck Surgery, Fatih Medicalpark Private Hospital, Istanbul, Turkey.*

**Keywords:** Mastoid, Nasal septum, Nasal surgical procedures, Temporal bone

## Abstract

**Introduction:**

The aim was to explore the developmental relationships between the angles of septal deviations and the degree of the mastoid pneumatization.

**Materials and Methods:**

In total, 143 patients with a diagnosis of septal deviation who underwent septoplasty were included. The patients were divided into three groups in terms of the angles of the septal deviation. The angle of the septal deviations was defined as mild (<9 degrees), moderate (between 9 and 15 degrees) and severe (15 degrees and above). The degree of the mastoid pneumatization of each groups were compared.

**Results:**

In right-sided septal deviation subjects, the right mastoid air cell volumes of group mild, moderate and severe were 6,31±2,33 cm3, 5,20± 1,51 cm3, and 5,31±1,57 cm3, respectively. The mean right mastoid volumes of each groups did not differ in right-sided deviations subjects (P>0.05). The mean left mastoid volumes of each groups did not differ in right-sided deviations subjects (P>0.05). In right-sided septal deviation subjects, the mean volume of the right and left-sided mastoid air cells of each groups did not differ (P>0.05). In left-sided septal deviation subjects, the mean volume of the right and left-sided mastoid air cells of each groups did not differ (P>0.05).

**Conclusions:**

No developmental relationships between the angles of septal deviations and the degree of the mastoid pneumatization was observed in the study.

## Introduction

Adequate knowledges of the degree of the mastoid pneumatization (MP) are substantial for otologist for several causes including the surgical-anatomical reasons, the choice of the surgical technique and the enlargement of mastoid cavity (1,2). The normal process of the MP starts in the perinatal period in the last weeks before birth. Pneumatization process of the mastoid bone progresses during infancy and childhood through to puberty. It is completely mature at approximately 15 years of age in males and ten years of age in females, although the development of MP varies between individuals (3,4).

Although the air cell system of mastoid bone (ACSMB) is propounded as an air cistern for the middle ear, knowledge of the physiologic functions of the ACSMB remains unclear. The development of the ACSMB starts from the antrum, but some peritubal and hypotympanic cells develop from the eustachian tube (ET) and hypotympanum, respectively (5,6).

The normal mucosa of the middle ear provides the normal process of the MP (7-9). Therefore, several factors are blamed for the development of the ACSMB in literature including such as chronic otitis media (COM), age, gender, genetic factors and environmental conditions (10-14). Also, the term of pneumatization is used in a pathogenetic involvement of mastoid bone (15). It has been propounded that the pneumatization of the ACSMB has been affected by the positive pressure in the nasopharynx through the ET (16). Considering the location of the ACSMB, anatomic variability of adjacent structures affect the development of the MP. Also, total nose airflow and the nasopharyngeal positive pressure are blamed for the development of the ACSMB (17). In study of Maier et al. reported that septal deviation has been altered the nasopharynx pressure (18). It is generally accepted that non-traumatic nasal septal deviation (NSD) is relatively rare condition in childhood and the most septal deviations occur in the period of growth which is intensive in puberty-when the mastoid pneumatization is almost finished. In literature, there have been few studies of investigating the relationship between the degree of the MP and the angles of the septal deviation. We address this topic in this study. We studied the relationships between the degree of the MP and the angles of the NSD. 

## Materials and Methods

We retrospectively evaluated data collected from May 2014 to January 2016 on patients treated at our hospital. Totally, 143 patients who underwent nasal corrections surgery due to a diagnosis of NSD were included. Patients with sinonasal polyps, chronic otitis media, chronic sinusitis, concha bullosa, below the age of 18 years, previous septoplasty or endoscopic sinus surgery history, sinonasal neoplasm, and history of maxillofasial trauma were excluded from the study. Paranasal sinus computed tomography (CT) imaging before septoplasty was performed in all patients. The side-effects of radiation were explained to all patients prior to CT, as was the reason why CT was planned. Local Ethics Committee approved the present study protocol (ethical committee number: 2016/34). 

**Fig 1:**
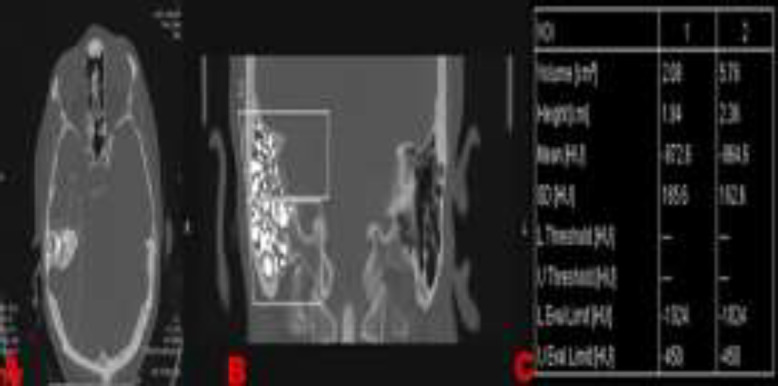
(**a**) The measurement of right mastoid air cell volume on the computed tomography (CT) axial slice with a gray-scale similar to air. (**b**) The measurement of right mastoid air cell volume on the CT coronal slice with a gray-scale level similar to air. (**c**) Calculation of the areas and the volumes of the extracted pneumatized parts of mastoid bone

ACSMB with a gray-scale level similar to air in the temporal bone were determined on the tomography imaging in this volumetric procedure. After image conducting, only the volumes of the extracted pneumatized portions were measured. Both sides were calculated separately in all patients (Fig.1a-c). Images were standardized for all patients by taking similar views of the basal turn of the cochlea, the internal acoustic canal, and the ossicular chain of either ear, in both axial and coronal slices. All patients have no pathologic findings on temporal bone slices.

**Fig 2:**
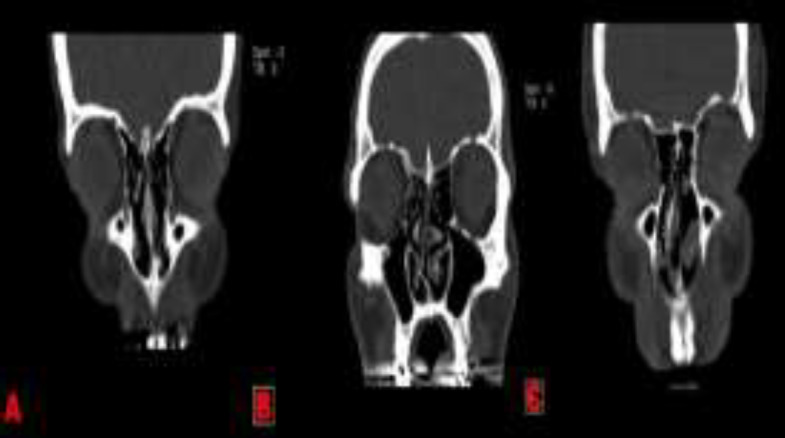
(**a**) The measurement of the angle of septal deviations for mild group. (**b**) The measurement of the angles of septal deviations for Moderate group. (**c**) The measurement of the angles of septal deviations for Severe group

The nasal septal angles were determined in coronal CT slices. The nasal septal angles were defined as the angles made by a straight line drawn along the line between the superior placement of the septum at the level of crista galli and the inferior placement of the septum at the level of maxillary crest where the line intersected the line from the superior placement of the septum at the level of crista galli to the apex of the NSD in the region of the ostiomeatal complex (Fig.2a-c).

All patients were separated into three groups in terms of the angles of the NSD. The angle of the NSD that previously described by Elahi et al. was defined as mild (<9 degrees), moderate (between 9 and 15 degrees) and severe (15 degrees and above) group. The degree of the MP of each groups were compared.

Statistical analysis

Number Cruncher Statistical System (NCSS) 2007 software (Kaysville, UT, USA) was used for all statistical analyses. Descriptive statistics were provided. The significance of each intergroup difference was analyzed using Student’s *t*-test, and the significance of any difference in median values was explored with the aid of the Mann-Whitney U-test. One-way analysis of variance (ANOVA) test and Tukey post hoc test were used to assess the differences between the angeles of the septal deviations and the degree of the mastoid volume. Pearson and Spearman correlations were calculated to explore the significance of differences between categorical variables. Qualitative data comparisons were performed using the Pearson χ^2^ test and the Fisher–Freeman–Halton test. 

## Results

We counted in 143 patients: 98 (68.5%) females and 45 (31.5%) males. The average age was 37.04 ± 15.38 years (range: 18-67 years). Groups properties are listed in (Table.1).

**Table 1:** Summary of group characteristics

Variables	Mild (n: 46)	Moderate (n: 46)	Severe (n: 51)
**Age (years)**	36.95 ± 14.48	37.12 ± 16.02	37.06 ± 12.24
**Gender**			
**-Females**	32 (69.5%)	30 (65.2%)	36 (70.6%)
**-Males**	14 (30.5%)	16 (34.8%)	15 (29.4%)
**The side septal deviation**			
**-Right**	19 (41.3%)	24 (52.2%)	27 (52.9%)
**-Left**	27(58.7%)	22 (47.8%)	24 (47.1%)

 The septal deviation deformities were determined on 70 right-sided (49.0%) and 73 left-sided (51.0%). The right-sided deviations include 19 mild, 24 moderate and 27 severe subjects. The left-sided deviations include 27 mild, 22 moderate and 24 severe subjects. In right-sided NSD subjects, the right ACSMB volumes of mild, moderate and severe groups were 6,31±2,33 cm^3^, 5,20±1,51 cm^3^, and 5,31±1,57 cm^3^, respectively. The mean right mastoid volumes of each groups did not differ in right-sided deviations subjects (P>0.05). In right-sided deviation subjects, the left ACSMB volumes of mild, moderate and severe group were 6,67±2,65 cm^3^, 5,32±1,66 cm^3^ and 5,51±1,58 cm^3^, respectively. The mean left mastoid volumes of each groups did not differ in right-sided deviations subjects (P>0.05) (Table. 2). In left-sided NSD subjects, the left ACSMB volumes of mild, moderate and severe group were 5,67±2,54 cm^3^, 5,77 ± 1,68 cm^3^ and 6,41 ± 2,47 cm^3^, respectively. The mean left mastoid volumes of each groups did not differ in left-sided deviations subjects (P>0.05). In left-sided NSD subjects, the right ACSMB volumes of mild, moderate and severe group were 5,87±2,07 cm^3^, 5,68±1,49 cm^3^, and 6,48±3,03 cm^3^. The mean right mastoid volumes of each groups did not differ in left-sided deviations subjects (P>0.05) (Table.3). In right-sided septal deviation subjects, the mean volume of the right and left-sided ACSMB of each groups did not differ (P>0.05). In left-sided NSD subjects, the mean volume of the right and left-sided ACSMB of each groups did not differ (P>0.05) (Table.4).

**Table 2:** Right and Left mastoid pneumatization in right-sided septal deviations

**Right-sided**		**Groups**			**Total**	^a^ **P**	**Post-hoc**		
		**Mild**	**Moderate**	**Severe**					
							**I-II**	**I-III**	**II-III**
Right Mastoid volume(cm^3^)	n	19	24	27	70	0.099	0,118	0,156	0,977
	Aver ± SD	6,31 ± 2,33	5,20 ± 1,51	5,31 ± 1,57	5,54 ± 1,83				
									
Left Mastoid volume (cm^3^)	n Aver ± SD	19 6,67 ± 2,65	24 5,32 ± 1,66	27 5,51 ± 1,58	70 5,76 ± 2,00	0.063	0,070	0,125	0,936

^a^ **P**, Oneway Anova Test; Aver = average; SD = standart deviation.

**Table 3:** Right and left mastoid pneumatization in left-sided septal deviations

**Left-sided**		**Groups**			**Total**	^a^ **P**	**Post-hoc**		
		**Mild**	**Moderate**	**Severe**			**I-II**	**I-III**	**II-III**
Right Mastoid volume (cm^3^)	n	27	22	24	73	0.469	0,955	0,620	0,475
	Aver ± SD	5,87 ± 2,07	5,68 ± 1,49	6,48 ± 3,03	6,01 ± 2,29				
									
Left Mastoid Volume (cm^3^)	n	27	22	24	73	0.475	0,987	0,486	0,613
	Aver ± SD	5,67 ± 2,54	5,77 ± 1,68	6,41 ± 2,47	6,41 ± 2,47				

^a^ **P**, Oneway Anova Test; Aver = average; SD = standart deviation.

**Table 4:** Comparison of right and left mastoid pneumatization in right-sided and left sided septal deviations

**Right-sided**		**Right**	**Left**	^a^ **p**
Mastoid pneumatization(cm^3^)	Aver±SD	5,54±1,83	5,76±2,00	0,297
Left-sided				
Mastoid pneumatization(cm^3^)	Aver±SD	6,01±2,29	5,94±2,28	0,714

^a^P, Paired Samples Test

Aver, average

SD, standart deviation.

## Discussion

The success of the middle ear surgery depends on the condition of middle ear cavity (MEC). Otherwise, the relationship between some otological diseases and the condition of the MEC was well documented in literature. However, the development of MP varies individually. In literature, two opinions have been propounded among interindividual variations of the degree of the mastoid pneumatization. Due to Diamant’s theory (15), the mastoid volume was appointed genetically and a small ACSMB volume predisposes to pathologic involvement of the middle ear cavity. Some authors supported his theory and explored the relationship between the MP and genetical diseases. In study of Sade et al. (10) reported that patients who were suffered from otosclerosis have larger temporal bone pneumatization than do healthy subjects. Similarly, Pata et al. (13) explored the relationship between presbycusis and MP considering the etiologies of both are reflected to have genetic factors. The cited authors found no differences between the presbycusis subjects and normal subjects considering the volume of MP.(13) In another study, Todd et al. (11) reported that MP of cystic fibrosis patients who have smaller frequency of otitis media than normal population was larger than the normal population (11). Due to Wittmaack’s opinion (19), normal mucosa is a necessary for the native MP process. Increasing the number of pathologic condition of the MEC among infancy decreases the degree of the MP. This theory is also called the environmental theory. Also, this theory legimitized to some authors. Considering the location of the ACSMB, variations of adjacent structures may affect the development of the MP. The most important anatomic structures are the ET, nasal and nasopharyngeal structures. In study of Apuhan et al. (20) noted that the size of adenoid tissue did not influence the development of the MP. In another study, Hindi et al. (21) reported a positive correlation between the MP and the pneumatization of the sphenoid sinus. Nasal pathologies lead to decreased nasal airflow and alter the nasopharynx pressure. In study of Maier et al. (18) noted that surgical correction of the severe nasal pathologies might be beneficial before the tympanoplasties. In another study, Koch et al. (22) deduced that negative middle ear pressure (MEP) in patients with impaired nasal ventilation can be recovered by surgery. Due to these reports, the presence of NSD affects the MEP negatively. Also, nasal pathologies should be improved before otological surgery. In study of Gencer et al. (23) investigated the associations of NSD on MP. The patients in their study were divided into three groups in terms of the angles of NSD. Their results indicated that severe NSDs significantly influence the ACSMB volume. In the present study, our results suggested that the angles of NSDs did not affect the degree of the MP. The MP was higher in group mild than other groups (albeit not significantly). The discrepancies among study of Gencer et al.(23) with our study may be attributable to the imaging parameters used, subject data and sample size. The measured mastoid volume was higher in the study of Gencer et al.(23) than the present study. Therefore, our results were not compatible with the study of Gencer et al.(23). In our study, we excluded the patients with COM. Hypothesis of our study was to evaluate the impact of the angle of NSD on the degree of MP. For this reason, affecting factors of the development of mastoid pneumatization were excluded. However, if the patients with COM was included the present study, it might affect the results. Also, we cannot determine the exact reason of developmental relationships between the angles of septal deviations and the degree of the MP. The small sample size and the lack of randomization were limited to the present study. However, we cannot assert that septoplasty may raise the success rate of otological surgery in patients with NSD considering the lack of the assessment of outcome of otological surgery. Our study was also a computer-assisted anatomic study. In conclusion, no developmental relationships between the angles of septal deviations and the degree of the MP was observed in the study. Further studies are obligatory to support these findings.
